# Liver Injury and Elevated FIB-4 Define a High-Risk Group in Patients with COVID-19

**DOI:** 10.3390/jcm11010153

**Published:** 2021-12-28

**Authors:** Dana Crisan, Lucretia Avram, Cristiana Grapa, Alexandra Dragan, Dan Radulescu, Sorin Crisan, Alin Grosu, Valentin Militaru, Elena Buzdugan, Laurentiu Stoicescu, Liliana Radulescu, Felix Ciovicescu, Delia Bunea Jivanescu, Oana Mocan, Bogdan Micu, Valer Donca, Luminita Marinescu, Antonia Macarie, Marina Rosu, Andrada Nemes, Rares Craciun

**Affiliations:** 1 Faculty of Medicine, “Iuliu Hatieganu” University of Medicine and Pharmacy, 400000 Cluj-Napoca, Romania; crisan.dc@gmail.com (D.C.); alexandra.dragan@elearn.umfcluj.ro (A.D.); dan.radulescu@umfcluj.ro (D.R.); Sorin.Crisan@umfcluj.ro (S.C.); alin.grosu@umfcluj.ro (A.G.); valentin.militaru@umfcluj.ro (V.M.); cristina.buzdugan@umfcluj.ro (E.B.); laurentiu.stoicescu@umfcluj.ro (L.S.); lili_m_radulescu@yahoo.com (L.R.); felix.ciovicescu@umfcluj.ro (F.C.); bunea.delia@umfcluj.ro (D.B.J.); oana.mocan@umfcluj.ro (O.M.); micu.vasile@umfcluj.ro (B.M.); ioan.donca@umfcluj.ro (V.D.); pasca.luminita@umfcluj.ro (L.M.); macarie.antonia@umfcluj.ro (A.M.); ROSU.MARINA@elearn.umfcluj.ro (M.R.); craciun.rares.calin@elearn.umfcluj.ro (R.C.); 2 Department of Internal Medicine, Clinical Municipal Hospital Cluj-Napoca, 400139 Cluj-Napoca, Romania; 3 Department of General Surgery, Clinical Municipal Hospital Cluj-Napoca, 400139 Cluj-Napoca, Romania; 4 Intensive Care Unit I, Cluj County Emergency Hospital, 400006 Cluj-Napoca, Romania; nemes.andrada.raluca@elearn.umfcluj.ro

**Keywords:** COVID-19, liver, FIB-4, mortality

## Abstract

Liver involvement in Coronavirus Disease 2019 (COVID-19) has been widely documented. However, data regarding liver-related prognosis are scarce and heterogeneous. The current study aims to evaluate the role of abnormal liver tests and incidental elevations of non-invasive fibrosis estimators on the prognosis of hospitalized COVID-19 patients. We conducted a retrospective cohort study to investigate the impact of elevated liver tests, non-invasive fibrosis estimators (the Fibrosis-4 (FIB-4), Forns, APRI scores, and aspartate aminotransferase/alanine aminotransferase (AST/ALT) ratio), and the presence of computed tomography (CT)-documented liver steatosis on mortality in patients with moderate and severe COVID-19, with no prior liver disease history. A total of 370 consecutive patients were included, of which 289 patients (72.9%) had abnormal liver biochemistry on admission. Non-survivors had significantly higher FIB-4, Forns, APRI scores, and a higher AST/ALT ratio. On multivariate analysis, severe FIB-4 (exceeding 3.25) and elevated AST were independently associated with mortality. Severe FIB-4 had an area under the receiver operating characteristic (AUROC) of 0.73 for predicting survival. The presence of steatosis was not associated with a worse outcome. Patients with abnormal liver biochemistry on arrival might be susceptible to a worse disease outcome. An FIB-4 score above the threshold of 3.25, suggestive of the presence of fibrosis, is associated with higher mortality in hospitalized COVID-19 patients.

## 1. Introduction

The systemic nature of Severe Acute Respiratory Syndrome Coronavirus 2 (SARS-CoV2) infection has been well documented since the COVID-19 pandemic ground the world to a halt in early 2020 [1]. In the subsequent months, a collective effort emerged, as numerous teams reported their experience in an attempt to sketch out the disease’s physiology, natural history, and potential therapeutic targets. In the meantime, the scientific community has achieved the improbable task of quickly developing multiple safe and effective vaccines. Yet, almost two years after being initially reported in Wuhan, the pandemic flame is far from being extinguished. Largely due to unequal vaccine availability, widespread anti-vaccination movements, and the emergence of new viral variants, the pandemic remains unpredictable. Thus, all additional information brought to light becomes extremely valuable.

The independent outcome predictor status of cardiovascular disease, metabolic syndrome, and diabetes mellitus on the course of COVID-19 was observed early and widely discussed [2,3,4]. Moreover, there are multiple other high-risk groups that are becoming increasingly better defined, such as immunocompromised patients (either transplant recipients or other groups on immunosuppressive medication) or patients with ongoing malignancy [5,6,7] However, the involvement of the liver either in the pathophysiology of COVID-19 or as an underlying risk factor was initially inconspicuous, even if approximately half of the patients had abnormal liver tests on admission [8]. In the latter months of 2020 and early 2021, however, multiple reports have been published on the role of abnormal liver workup, especially in patients with undocumented liver disease, fibrosis, and/or quiescent Metabolic Associated Fatty Liver Disease (MAFLD) and undiagnosed Non-Alcoholic Steatohepatitis (NASH).

The largest part of liver-related clinical research (excluding portal hypertension) in COVID-19 consists of two subtopics: the relevance of liver test abnormalities during hospitalization and the role of MAFLD as an outcome predictor. As per se abnormal tests have little relevance out of context, the first matter converged towards the role of non-invasive scores in assessing various outcomes, such as disease severity, hospitalization, and, ultimately, mortality. Among the most useful tools, the FIB-4 score has gained the most traction. Initially developed as a simple four-variable tool to estimate fibrosis in HCV/HIV co-infected patients [9] and further validated on a wider range of etiologies, including MAFLD [10], FIB-4 has hinted towards applicability in COVID-19, as some recently published papers suggest [11,12,13,14]. Along with FIB-4, there are multiple other easy to use non-invasive scores, such as FORNS [15], APRI [16], and the AST to ALT ratio [17]. These scores have extensive validation in other liver disease etiologies and might extend their applicability in the setting of COVID-19.

On the other hand, the role of MAFLD has not been clearly established. MAFLD is a common finding in patients with metabolic syndrome and/or diabetes mellitus, which are clear risk factors for a severe COVID-19 disease course and mortality. Classifying MAFLD patients as high-risk for a poor outcome can be grounded on the assumption that an intricate interrelation between obesity, vitamin D deficiency, and a chronic low-grade pro-inflammatory status leads to an ill-adapted immune response [18]. However, while MAFLD plus fibrosis (highly suggestive of NASH) appears to be associated with a worse outcome [13,19], there is conflicting data with regards to the role of quiescent MAFLD or incidental findings of steatosis in predicting definite endpoints [13,20,21].

Moreover, in the reorganized hospital settings, the accessibility of high-precision diagnostic tools has substantially decreased during the pandemic. Techniques such as transient elastography or high-performance ultrasound machines with pre-installed shear-wave elastography and steatosis assessment applications remained underused, as care moved closer to the bedside. This inconvenience further places the burden on quick, low-resource-consuming tools to provide essential diagnostic and prognostic data.

### Aims

Our current study had the major objective of establishing the role of abnormal liver tests and incidental findings of fibrosis on admission, assessed by FIB-4, FORNS, APRI, AST/ALT in predicting disease outcome in patients with undocumented pre-existing liver disease hospitalized for SARS-CoV2 infection. A secondary objective, in tight conjunction with the first one, was to evaluate the role of incidental findings of liver steatosis, diagnosed on admission at the thoracic CT scan, on disease course, intensive care unit admission, and mortality.

## 2. Materials and Methods

### 2.1. Study Design and Participants

The current research was designed as an observational, retrospective, longitudinal study. Patient enrollment took place in a single tertiary-care hospital, which has been transformed into a regional COVID-19-dedicated center. A consecutive series of patients was included between January 2021 and April 2021. All patients tested positive for SARS-CoV-2 RNA following a nasopharyngeal swab analyzed by real-time reverse-transcriptase polymerase chain reaction (rRT-PCR). None of the patients were vaccinated against SARS-CoV-2.

Per regional protocol, asymptomatic and mildly symptomatic patients with no additional risk factors (young, no documented associated conditions, negative thoracic CT scan) were not hospitalized, and thus, not included in our analysis. The rationale for not including such patients in our study was based on the intention to avoid skewing the dataset towards the less severe end of the disease spectrum, to avoid overestimating the effect size of the predictive analysis. Patients with previously diagnosed liver disease were excluded: infection with hepatitis B virus (HBV), untreated infection with hepatitis C virus (HCV), treated HCV infection with documented fibrosis, alcoholic liver disease or alcohol misuse disorder, biopsy-proven NASH, autoimmune hepatitis, primary biliary cholangitis, primary sclerosing cholangitis, hemochromatosis, Wilson’s disease, vascular liver disease, documented cirrhosis or clinically significant portal hypertension of any etiology, with or without any prior decompensation. The risk for drug-induced liver injury was assessed by analyzing chronic and recent medication on arrival. When found, patients on the drugs most frequently associated with liver injury were excluded. Patients with previously diagnosed myopathies, recent trauma, hematological conditions, and transplant recipients were also excluded.

### 2.2. Baseline Evaluation

Demographic data, as well as a comprehensive laboratory workup, were performed either in the emergency department or during day 1 of hospitalization. The laboratory workup consisted of a full blood count, inflammatory syndrome assessment (C-reactive protein (CRP), procalcitonin, D-dimer, fibrinogen, lactate dehydrogenase (LDH) and ferritin), liver function tests (aspartate aminotransferase (AST), and alanyl aminotransferase (ALT), total and direct bilirubin, GGT, alkaline phosphatase (ALP), albumin), coagulation (prothrombin time, international normalized ratio (INR), activated partial thromboplastin time (aPTT)), kidney function (urea, creatinine, electrolytes), and metabolic profile (fasting blood glucose, triglyceride levels, total cholesterol, HDL and LDL cholesterol levels, total protein levels). All patients with liver test abnormalities were screened for HBV and HCV infection, using HBs antigen and HCV antibodies, respectively.

A non-contrast thoracic computed tomography scan was performed on admission. The total severity score (TSS) was used to assess COVID-19 lung involvement, as described by Li K. et al. [22]. Patients were classified as having a severe form of the disease if the TSS score exceeded 8. Hepatic steatosis was assessed using a previously validated protocol [13]. CT scans were interpreted by a single radiologist. The diagnosis of liver steatosis was based upon two major criteria: an attenuation coefficient of less than 40 Hounsfield Units (HU) in a 20 cm^2^ area in segments VII and VIII and an attenuation coefficient above 10 HU in a 5 cm^2^ area in the splenic parenchyma compared to the previously described area in the liver. A liver/spleen ratio cut-off value of 0.7 was used to discriminate patients with severe steatosis.

### 2.3. Non-Invasive Scores

All scores were calculated using the laboratory data obtained on admission, as originally described. Cut-off values were used to define high-risk groups for the presence of advanced steatosis, according to the initial validation studies. To account for the low specificity of FIB-4 in elderly patients (age group > 65), a cut-off of 2 was used, as previously described [23]. Calculation formulas and cut-off values are depicted in the table below (Table 1).

### 2.4. Follow-Up

Patients were followed during their hospitalization, recording total hospital stay, admission to the intensive care unit (ICU), and death.

### 2.5. Ethical Considerations

By design, the study complies with all current ethical considerations. The protocol was approved by the Institutional Ethics Committee of the Clinical Municipal Hospital in Cluj-Napoca. Informed written consent was obtained from each patient included in the study and the study protocol was developed under the ethical guidelines of the modified 1975 Declaration of Helsinki.

### 2.6. Statistical Analysis

Continuous variables were expressed as either mean ± standard deviation (SD) or median and 95% confidence interval (CI), for normal and non-normal distributions, respectively. Student’s *t*-test was used for the comparison of normally distributed variables, while the Mann–Whitney U test was used for non-normal variables. The chi-square test was used for categorical variables. Multivariate analysis was designed to minimize model overfitting. Kaplan–Meyer curves with the log-rank test were used for survival analysis. The threshold for statistical significance was set at 0.05. The discriminative abilities of different variables were analyzed using the receiver operating characteristic (ROC) curve. Statistical analysis was performed by a certified biomedical statistician using SPSS software, version 28.0 (SPSS Inc., Chicago, IL, USA).

## 3. Results

### 3.1. Baseline Characteristics

Our cohort consisted of 370 patients with confirmed SARS-CoV-2 infection. The median age was 65.5 years old (58.11–69.3), with a slight male predominance (*n* = 220, 59.49%). More than half of the patients (51.6%) had severe COVID-19 according to the TSS score (TSS > 8). The most common co-morbid condition was arterial hypertension (*n* = 217, 58.8%), followed by type 2 diabetes mellitus (*n* = 90, 24.3%). None of the patients had a prior history of chronic liver disease.

Throughout hospital stay, 289 (72.94%) patients developed a form of liver injury, defined by AST and ALT elevations, and 165 (50.92%) developed a cholestatic pattern with both ALP and GGT elevated. On admission, median AST and ALT levels were 47.5 (47.26–93.33) and 38 (33.24–77.17), while the peak values during hospitalization were 69.5 (65.82–130.28) and 86.5 (84.66–220.94), respectively. As assessed by analyzing liver parenchyma densities on the index CT scan, the prevalence of liver steatosis was 39.5% (*n* = 145). Regarding the non-invasive tests used for the diagnosis and staging of liver fibrosis, FIB-4 had a median value of 2.6 (2.1–4.71), AST/ALT 1.51 (1.26–1.84), APRI 0.42 (0.4–1.13) and Forns 6.61 (5.78–7.11). Severe fibrosis according to FIB-4 was found in 109 (29.5%) patients and 156 (42.2%) after adjusting the score for age > 65, according to guideline recommendations. The baseline characteristics of our cohort are summarized in Table 2.

### 3.2. Univariate Outcome Analysis

A univariate analysis was performed to assess the variables associated with in-hospital mortality. Of the 370 total patients, 43 (11.6%) have died during hospitalization. Non-survivors had a significantly higher TSS score, were older, had a more pronounced pro-inflammatory status (as derived from higher levels of CRP, procalcitonin, ferritin, fibrinogen, D-dimers, and LDH), and had worse kidney function. Non-survivors had significantly lower platelet counts. Among liver function tests, on admission AST and peak AST levels were significantly higher in the latter group. According to CT scans, 20 (46.51%) patients in the non-survivor group and 126 (38.53%) in the survivor group were diagnosed with liver steatosis, yet with no statistical difference. Concerning the fibrosis scores, FIB-4 (*p* < 0.005) and the prevalence of severe fibrosis (as assessed by FIB-4 cut-offs) were significantly higher in the non-survivor group (*p* = 0.04). All the other scores, namely AST/ALT (*p* < 0.001), APRI (*p* = 0.009), and Forns (*p* = 0.001) were also significantly associated with a worse outcome, with higher values in non-survivors. The differences between the two groups are summarized in Table 3.

The association between the values of FIB-4, APRI, Forns, AST/ALT exceeding the threshold for a severe score and the presence of liver steatosis was analyzed (Table 4). While severe FIB-4 was not associated with a higher prevalence of steatosis, high age-adjusted FIB-4 (subgroup of patients over 65 years old, *p* = 0.01), Forns (*p* < 0.01), APRI (*p* = 0.03) and AST/ALT (*p* = 0.02) scores were more frequently encountered in patients with steatosis. As a matter of nuance, liver enzyme elevations (AST, ALT or both) were more frequently associated with higher scores across the board, suggestive of the presence of fibrosis. However, this relationship was also valid for the Forns score, which does not include AST or ALT in its calculation formula.

We investigated whether the presence of MAFLD (detected via the index CT scan) had any prognostic relevance. No significant associations were found between liver steatosis and COVID-19 severity (*p* = 0.61), hospital stay (*p* = 0.11), ICU admission (*p* = 0.23), or survival (*p* = 0.56).

### 3.3. Multivariate Analysis

**Figure 1:**
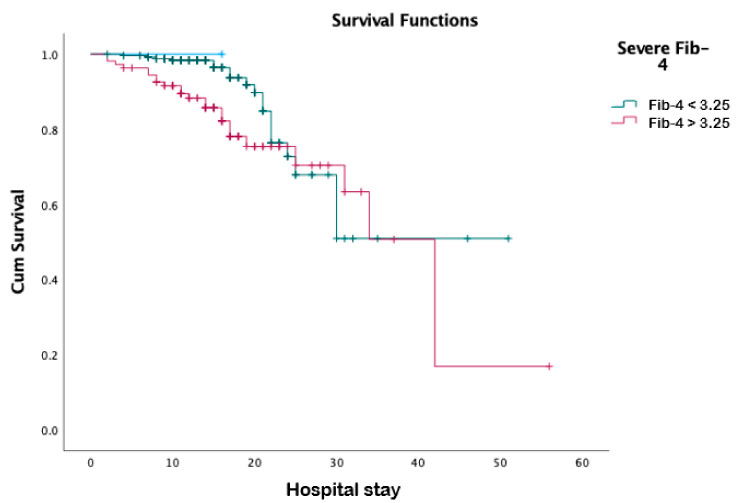
Survival analysis for severe FIB-4 score, defined as FIB-4 > 3.25.

All the non-invasive scores were included in a multivariate analysis to assess their predictive value for survival. Among them, only FIB-4 and elevated AST values were significantly associated with poor survival (*p* = 0.046 and *p* = 0.037, respectively) (Table 5). Using the conventional cut-off value for high FIB-4 (3.25), the Kaplan–Meyer survival analysis revealed that an FIB-4 exceeding the threshold was associated with a worse survival (*p* = 0.05) (Figure 1). A similar correlation was found for age-adjusted FIB-4 (*p* = 0.036) and Forns (*p* < 0.001).

**Figure 2:**
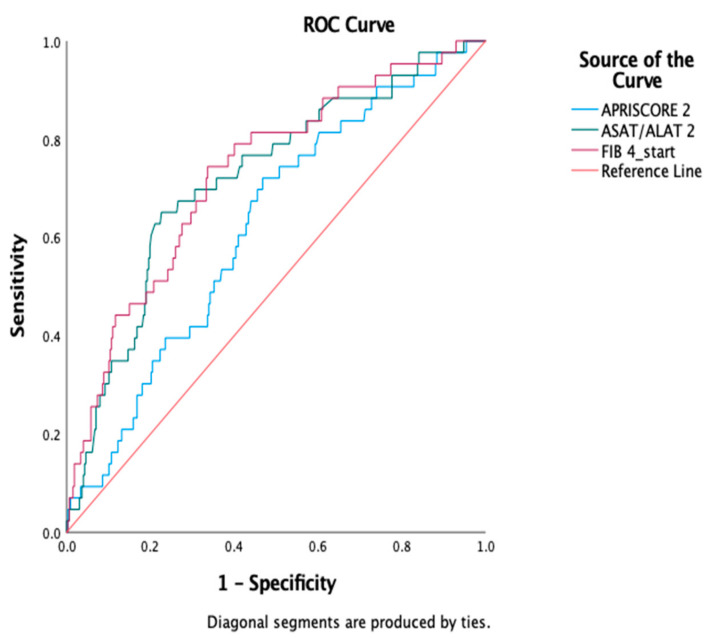
AUROC curves comparison for FIB-4, AST/ALT, APRI scores.

We also analyzed the AUROC curves for the fibrosis scores (Figure 2) and found that FIB-4 had the best predictive value for survival, with an area under the curve (AUC) of 0.731, while AST/ALT was a close second, with an AUC of 0.72, both suggestive of good discriminatory performance. The Forns score had an AUC of 0.679, while APRI had the lowest AUC of 0.622.

## 4. Discussion

In response to our major objective, the results suggest that abnormal liver enzyme levels and high non-invasive scores in assessing the risk of advanced fibrosis are associated with a worse in-hospital outcome. We found a very high prevalence of abnormal liver biochemistry tests. Over two-thirds of our cohort (>72%) had elevated transaminases, while more than half of the patients (50.92%) also had cholestasis. These figures are significantly higher than previously published reports, which place the prevalence of liver test abnormalities between 14 and 53% [8]. However, given the nature of our design, asymptomatic patients or those on the less severe end of the disease spectrum were not included in our cohort. Therefore, the balance was skewed towards moderate and severe forms, with a higher prevalence of systemic injury. Hepatic involvement is thought to be related to direct viral infection of the liver cells, sepsis, or drug hepatotoxicity [24]. Since patients presented with high AST and ALT values at admission in our cohort, drug hepatotoxicity seems unlikely. Emerging data have shown that the SARS-CoV-2 virus can have a direct cytopathic effect on hepatocytes and cholangiocytes, with autopsy reports detecting viral material in the liver tissue in up to 41% of cases. The histological pattern is unspecific, appearing to be similar to drug-induced liver injury or sepsis. The immune response can also be dysregulated, leading to the activation of cytokines and subsequent hepatic inflammation [25]. The high number of patients that presented with elevated liver enzymes might therefore be indicative of the direct infection of the liver cells. However, the relevance of incidental findings of elevated transaminases or cholestasis on disease outcome is to this point unclear, as the liver might be only a quiescent collateral victim of systemic inflammation and viral ubiquity in the absence of overt liver failure. Our study found no statistical difference between liver enzyme values at admission between survivors and non-survivors. On the other hand, peak levels of AST were significantly higher in non-survivors, suggesting that liver injury might be associated with the risk of death, which is in concordance with other studies [26].

Preliminary reports have additionally proved that pre-existing liver diseases lead to worse outcomes for COVID-19 patients [27,28,29]. However, in our group, we excluded patients with previously diagnosed liver diseases and screened for the most common causes for hepatic injury, by testing for hepatitis B and C. However, the impact of subclinical or unrecognized liver disease in COVID-19 patients is still unknown, and the most common culprit is MAFLD. In this light, a simple, reliable score that could help clinicians categorize patients who need further assessment may be of great help in planning a subsequent liver-related work-up.

There is a wide range of non-invasive tests designed to rule in or out chronic liver disease and, more specifically, the presence of advanced fibrosis. Their perks and pitfalls have been elegantly addressed in the most recent EASL position paper on the topic, published in the summer of 2021 (quote in commentary). Nevertheless, beyond their well-defined indications in the setting of previously documented liver disease or at-risk liver disease populations, the role of these non-invasive tests has been tested in more unorthodox scenarios. Their use has not necessarily been as indicators of advanced fibrosis, but rather as predictive factors for liver-related outcomes. In this framework, recent studies have suggested that FIB-4 could have a potential prognostic role in COVID-19 [11,14,30,31]. Our study further augments this idea since in our group of patients, presumably without any underlying liver disease, this score was significantly associated with the risk of death, with an AUROC of 0.74, the highest compared to other liver fibrosis scores. Furthermore, besides a high AST value, FIB-4 levels were the only ones that retained statistical significance in the multivariate analysis (*p* = 0.05). We opted for FIB-4 because of its simplicity and high validation in different liver etiologies [32]. We also applied other fibrosis scores, such as AST/ALT, APRI, and Forns, which had a limited predictive power.

As expected, high non-invasive scores (FIB-4 > 65, Forns and APRI) exceeding the cut-offs for advanced fibrosis, were all correlated with steatosis. Furthermore, high AST and ALT values (>50 UI/l), were also significantly associated with severe FIB-4 > 65 and Forns. While the association between FIB-4 and high transaminase levels was expected since the calculation formula includes AST and ALT (which are frequently elevated in coronavirus patients), their correlation with the Forns score was not a given. This key aspect might support the assumption that liver injury expands beyond incidental laboratory findings of elevated liver enzymes that can artificially alter non-invasive scores above conventional cut-offs, although the exact mechanisms are poorly defined. As proof, the Forns score was also statistically associated with survival prediction using the Kaplan–Meyer estimator (*p* < 0.001), which raises the question of whether non-invasive fibrosis evaluation should be performed for every patient, given their prognostic role in COVID-19.

The reason why some fibrosis scores’ elevation, mainly FIB-4, is related to COVID-19 outcome is still unclear. Samaniego et al. [11] pointed out the prognostic role of FIB-4 in 160 patients diagnosed with SARS-CoV-2 infection and proceeded to calculate the FIB-4 score in a subgroup (15% of the total), retrieving liver tests performed within six months of the diagnosis. They discovered that while AST and ALT levels were significantly higher at the time of COVID-19 infection, levels of FIB-4 were not significantly different. They concluded that high FIB-4 (>2.67) as a marker for advanced liver fibrosis could have a prognostic role for coronavirus patients. Their explanations reside from the fact that chronic liver disease is associated with a degree of baseline systemic inflammation that adds to COVID-19′s inflammatory response. Our study further nuances the matter of fibrosis scores being elevated. As mentioned above, a high FIB-4 could be explained by high levels of liver enzymes, which might be a result of cytopathic virus-related liver injury or systemic inflammation. On the other hand, other scores, like Forns, are not calculated using transaminase values, yet they still reach levels compatible with advanced fibrosis. This prompts a further question: are these scores truly indicative of histological fibrosis or do they reflect a type of liver injury associated with a worse outcome through other means? Their validation and role should be further explored in larger cohorts and additionally correlated with elastography tests. Unfortunately, in the context of COVID-19, these designs are difficult to implement, especially in high-disease-burden settings.

Another study on 70 patients with different hematological malignancies showed that FIB-4 > 3.85 was an independent predictor for mortality in patients with COVID-19 infection; the authors point out that the synergistic effect of SARS-CoV-2 virus on the liver and intrinsic hematopoietic abnormalities found in this subcategory of patients led to higher FIB-4 values, although admittedly their sample size was small [33]. Furthermore, a meta-analysis revealed that high FIB-4 values are associated with mortality in COVID-19 patients, thus augmenting the idea that this simple score used for detection of liver fibrosis and can be repurposed for predicting clinical outcome in COVID patients [32].

Globally, MAFLD prevalence is estimated at 24% of the population [34] and in COVID-19 patients, its prevalence is approximately 30% [35]. In our study, 146 (39.5%) patients had steatosis, diagnosed through CT imaging. The relatively higher percentage might be explained by the inclusion of patients with a more severe form of COVID-19 who required hospitalization, therefore who had a higher burden of co-morbid conditions associated with a metabolic imbalance and, consequently, MAFLD.

Studies that have evaluated MAFLD patients have related this disease to poor COVID-19 outcomes [36,37]. The exact mechanism is still unknown, although the proposed hypothesis revolves around the idea that steatosis leads to increased production of proinflammatory cytokines, thus exacerbating the cytokine storm related to SARS-CoV-2 infection [38,39]. Furthermore, MAFLD patients with high NFS (NAFLD fibrosis score) and high FIB-4 were associated with a higher risk of severe COVID-19 disease, even after adjusting for obesity and diabetes [40]. Not least, there is purported evidence of a high susceptibility to numerous infections, which typically follow a more severe disease course when compared to non-MAFLD patients. The implications range from recurrent and more severe bacterial pneumonia, urinary tract infections and urolithiasis, *Clostridoides difficile* colitis, and even more frequent and severe complications of *Helicobacter pylori* infection [18]. Our study assessed the impact of imaging diagnosed MAFLD disease on hospital stay, risk of death, and severity of the disease (according to TSS score). However, no statistically significant associations were found. The results comply with another study on a cohort of 539 patients that did not find a positive association between MAFLD and disease severity, mortality, or risk of progression [21]. However, some of these studies were limited by the fact that they used the HSI (hepatic steatosis index) score, which could have overestimated the presence of MAFLD [2]. Jin D. et al. pointed out that these conflicting results are additionally influenced by the different methods of establishing COVID-19 severity: either based on the need for mechanical ventilation, ICU admission, or on the premise that patients who did not need oxygen supplementation and could safely be home managed do not represent severe cases. The strength of our study resides in the fact that we established a diagnosis of steatosis based on CT evaluation, which, as mentioned, many of the studies involving either MAFLD or fibrosis scores lack. Additionally, we excluded the most common viral diseases (hepatitis B and C) as the underlying causes of previous liver disease.

We acknowledge that our study has multiple limitations, some of which are the direct result of conducting a clinical study within the barriers of a COVID-19 hospital setting. One such limitation was using fibrosis scores without histological assessment for direct comparison. However, we believe that assessing liver histology through liver biopsy in the context of COVID-19 was not a primary objective and might have harbored little clinical relevance for the purpose of our study. Furthermore, our cohort size was relatively modest. With regards to the use of non-invasive scores, there is not yet an established cut-off point for defining high FIB-4 values in the COVID-19 population. We used the 3.25 cut-off, typically utilized to rule out advanced fibrosis in high-risk patients with MAFLD, given the relatively high burden of steatosis in our cohort, in spite of some studies using other values. We decided against calculating our own cut-off, considering that it would have further complicated the already heterogeneous literature on the topic. Another important issue regards the reason for this particular score to be elevated, since in our group of patients, without any liver diseases and regardless of steatosis, it was still associated with the risk of death. Large cohort studies demonstrated that fibrosis scores such as FIB-4 also have limitations in the general population [41] so our result must be interpreted in the COVID-19 clinical scenario. However, in this context, FIB-4 may be elevated and considered a predictor for mortality regardless of any subclinical or undiagnosed liver disease. Further research that explains the pathogenic mechanism behind the correlation of high FIB-4 levels and risk of death in COVID-19 patients is needed.

## 5. Conclusions

Given the impact of the ongoing pandemic on the healthcare system and the expected bleak effect from deferring or cancelling treatments for chronic illnesses and delaying diagnosis for acute conditions, every datum on developing a rapid diagnosis for patients is crucial. Whether it is about recognizing an undiagnosed liver disease or predicting a severe outcome for COVID-19 patients, we demonstrated that fibrosis scores like FIB-4 could aid clinicians to categorize patients regarding prognosis and approach them correspondingly, and maybe even prioritize them for vaccination since they represent a group with a worse prognosis.

## Figures and Tables

**Table 1:** Non-invasive fibrosis scores.

Non-Invasive Score	As Originally Described in:	Formula	Cut-Off Values for High Risk of Advanced Fibrosis
FIB-4	Sterling R. et al. [9]	Age (years) × AST (IU/L)/platelet count (×10^9^/L) × √ALT (IU/L)	3.25 if age < 65 2.0 if age ≥ 65
AST/ALT ratio	Giannini E. et al. [17]	AST/ALT	0.8
Forns score	Wai C. et al. [15]	7.811 − 3.131 × ln (platelet count) + 0.781 × ln (GGT) + 3.467 × ln (age) − 0.014 × (total cholesterol)	6.9
APRI score	Lin Z. et al. [16]	AST/upper limit of normal)/platelet count (expressed as platelets × 10^9^/L) × 100	1

**Table 2:** Baseline characteristics.

Variable	Median (Confidence Interval)/No (%)
Age (years)	65.5 (58.11–69.3)
Gender (M, %)	220 (59.45%)
Biological parameters	
Hemoglobin (g/dL)	13.05 (11.54–13.96)
White blood cells (×10^9^/L)	7.05 (6.96–9.43)
Lymphocytes (×10^9^/L)	1.09 (0.93–1.36)
Neutrophils (×10^9^/L)	5.42 (5.52–7.57)
Platelets (×10^9^/L)	207.5 (193.04–266.21)
Random blood glucose (on admission, mg/dL)	126 (119.94–172.64)
C–reactive protein (mg/L)	139.5 (104.68–169.61)
Procalcitonin (ng/mL)	0.14 (−0.22–1.47)
Fibrinogen (mg/dL)	715.65 (682.69–941.94)
D-dimers (mg/dL)	529.45 (489.79–1333.77)
Ferritin (mg/dL)	824.1 (763.21–1808.79)
Lactate dehydrogenase (U/L)	697.5 (515.86–840.3)
Total cholesterol (mg/dL)	155.5 (142.65–177.42)
Triglycerides (mg/dL)	144 (130.79–198.37)
HDL-cholesterol (mg/dL)	32.5 (31.1–41.4)
Creatinine (mg/dL)	1.02 (0.76–2.33)
Urea (mg/dL)	43 (39.04–64.99)
Na (mmol/L)	139 (136.36–139.73)
K (mmol/L)	4.31 (4.23–4.69)
Creatinine clearance (MDRD) (mL/min/1.73 m^2^)	64.47 (52.26–78.74)
INR	1.07 (1–1.59)
Prothrombin Time (s)	17.85 (16.05–24.7)
Liver tests	
AST (U/L)	47.5 (47.26–93.33)
ALT (U/L)	38 (33.24–77.17)
AST and ALT elevations (N, %)	289 (72.94%)
ALP (U/L)	67.5 (63.17–92.24)
GGT (U/L)	91 (71.18–134.9)
ALP and GGT elevations (N, %)	165 (50.92%)
Bilirubin (mg/dL)	0.45 (0.41–0.66)
Fibrosis scores	
AST/ALT value	1.51 (1.26–1.84)
Severe AST/ALT	163 (44.1%)
APRI value	0.42 (0.4–1.13)
Severe APRI	88 (23.8%)
FIB-4 value	2.6 (2.1–4.71)
Severe FIB-4	109 (29.5%)
Age adjusted severe FIB-4	156 (42.2%)
Forns value	6.61 (5.78–7.11)
Severe Forns	199 (53.8%)
Disease severity and personal history	
TSS start	11.5 (10.44–13.31)
TSS maxim	11.5 (10.58–13.75)
Severe COVID *	191 (51.6%)
Liver steatosis on CT	146 (39.5%)
Arterial hypertension	217 (58.6%)
Ischemic heart disease	62 (16.8%)
Atrial fibrillation	38 (10.3%)
Heart failure	42 (11.4%)
Chronic obstructive pulmonary disease	34 (9.2%)
Stroke	25 (6.8%)
Chronic kidney disease	44 (11.9%)
Type 2 diabetes mellitus	90 (24.3%)
Neoplasia	27 (7.3%)

Abbreviations: ALT—alanine aminotransferase; AST—aspartate aminotransferase; ALP—alkaline phosphatase; GGT—gamma glutamyl transferase; CRP—C reactive protein; LDH—lactate dehydrogenase; TG—triglycerides; LDL—low density lipoproteins; HDL—high density lipoproteins; INR—international normalized ratio; PT—prothrombin time; CT—computer tomography; COPD—Chronic obstructive pulmonary disease; CVA—cerebrovascular accident; T2DM—type 2 diabetes mellitus; * Severe COVID according to TSS scoring (TSS > 8). Variables are shown as median (95% confidence interval).

**Table 3:** Univariate comparison between survivors and non-survivors.

Variable	Survivors (*n* = 327, 88.4%)	Non-Survivors (*n* = 43, 11.6%)	*p*
Age (years)	62 (55.4–67.5)	73 (60.53–89.47)	<0.001
Gender (M, %)	190 (86.36%)	30 (69.76%)	0.096
Biological parameters			
Hemoglobin (g/dL)	13.05 (11.24–14.04)	13 (9.5–17.09)	0.006
White blood cells (×10^9^/L)	7.45 (6.96–9.74)	6.22 (2.59–12.35)	0.23
Lymphocytes (×10^9^/L)	1.09 (0.94–1.40)	0.85 (0.00–2.05)	<0.001
Neutrophils (×10^9^/L)	5.57 (5.47–7.79)	5.42 (2.19–10)	0.017
Platelets (×10^9^/L)	231 (206–285)	153.5 (72.98–227.52)	0.035
Random blood sugar (on admission, mg/dL)	125 (116.01–178.69)	128 (81.31–200.69)	0.026
C reactive protein (mg/L)	132.29 (92.6–169.65)	169.5 (118.35–216.14)	<0.001
Procalcitonin (ng/mL)	0.13 (0.01–1.68)	0.25 (0.01–1.3)	0.001
Fibrinogen (mg/dL)	746.2 (677.67–989.72)	710.15 (677.65–733.19)	0.015
D-dimers (mg/dL)	529.45 (461.6–934.43)	1871.15 (−1285.41–5246.66)	0.04
LDH (U/L)	627 (481.46–874.93)	698.5 (500.33–854.66)	<0.001
Ferritin (mg/dL)	742.75 (696.38–1956.5)	1073.5 (358.78–1808.81)	0.008
Cholesterol (mg/dL)	159.5 (142.76–184.04)	140.5 (119.17–167.32)	0.001
TG (mg/dL)	144 (128.5–208.69)	140.5 (74–215)	0.557
HDL (mg/dL)	31.5 (29.23–40.87)	38.5 (26.06–58.44)	0.881
LDL (mg/dL)	100 (83.74–121.96)	71 (58.54–85.96)	0.014
Creatinine (mg/dL)	0.96 (0.53–2.36)	1.75 (0.07–4.23)	<0.001
Urea (mg/dL)	38.5 (34.76–51.98)	90.5 (22.21–168.28)	<0.001
Na (mmol/L)	139 (135.93–139.77)	138.5 (133.05–144.95)	0.115
K (mmol/L)	4.41 (4.25–4.72)	3.95 (3.01–5.67)	0.008
Creatinine clearance (MDRD) (mL/min/1.73 m^2^)	67.62 (55.16–83.85)	40.39 (3.38–94.37)	<0.001
INR	1.07 (0.96–1.68)	1.14 (0.92–1.37)	0.004
Prothrombin Time (s)	17.85 (15.18–25.58)	18.75 (12.73–27.96)	0.008
Liver tests			
AST (U/L)	51.02 (46.97–55.07)	64.55 (49.76–79.35)	0.04
ALT (U/L)	50.45 (45.05–55.86)	38.95 (27.58–50.31)	0.13
ALP (U/L)	71.5 (65.64–87.76)	52.5 (−35.33–200.83)	0.212
GGT (U/L)	91 (70.62–141.78)	78 (−42.55–217.05)	0.201
Bilirubin (mg/dL)	0.44 (0.38–0.67)	0.63 (0.18–1.02)	0.082
Peak AST (U/L)	69.89 (63.56–76.21)	186.24 (71.84–300.64)	<0.001
Peak ALT (U/L)	118.85 (103.86–133.85)	153.13 (87.82–218.44)	0.91
Fibrosis scores			
AST/ALT	1.21 (1.12–1.30)	1.97 (1.41–2.53)	<0.001
Severe fibrosis depending on AST/ALT (*n*, %)	255 (78.7%)	36 (94.7%)	0.02
APRI	0.61 (0.51–0.71)	0.90 (0.51–1.29)	0.01
Severe fibrosis depending on APRI (*n*, %)	34 (10.5%)	7 (18.4%)	0.14
FIB-4	2.60 (2.29–2.90)	5.38 (3.32–7.44)	<0.001
Severe fibrosis depending on FIB-4 (*n*, %)	162 (50.2%)	30 (78.9%)	0.001
Forns	7.86 (7.57–8.15)	9.29 (8.60–9.98)	<0.001
Severe fibrosis depending on Forns (*n*, %)	130 (66.7%)	30 (96.8%)	<0.001
Disease severity and personal history			
Liver steatosis on CT	126 (38.53%)	20 (46.51%)	0.314
TSS start	11 (10.15–13.35)	12.5 (6.62–18.38)	0.004
TSS maxim	11 (10.28–13.72)	12.5 (5.77–20.23)	<0.001
Arterial hypertension	183 (56.5%)	28 (73.7%)	0.04
Ischemic heart disease	45 (13.9%)	12 (31.6%)	0.005
Atrial fibrillation	30 (9.3%)	6 (15.8%)	0.20
Chronic heart failure	31 (9.6%)	10 (26.3%)	0.002
Chronic obstructive pulmonary disease	23 (7.1%)	10 (26.3%)	<0.001
Stroke	22 (6.8%)	3 (7.9%)	0.79
Chronic kidney disease	30 (9.3%)	12 (31.6%)	<0.001
Type 2 diabetes mellitus	75 (23.1%)	13 (34.2%)	0.13
Neoplasia	21 (6.5%)	5 (13.2%)	0.13

Abbreviations: ALT—alanine aminotransferase; AST—aspartate aminotransferase; ALP—alkaline phosphatase; GGT—gamma glutamyl transferase; CRP—C reactive protein; LDH—lactate dehydrogenase; TG—triglycerides; LDL—low density lipoproteins; HDL—high density lipoproteins; INR—international normalized ratio; PT—prothrombin time; CT—computer tomography; COPD—Chronic obstructive pulmonary disease; CVA—cerebrovascular accident; T2DM—type 2 diabetes mellitus. Variables are shown as median (95% confidence interval).

**Table 4:** Association between fibrosis severity, steatosis, and markers of liver injury.

Fibrosis Scores	Associations	*p*
Severe FIB-4	Steatosis	0.721
	ALT > 50	0.196
	AST > 50	<0.001
Severe FIB-4 > 65	Steatosis	0.012
	ALT > 50	<0.001
	AST > 50	0.008
Severe Forns	Steatosis	0.001
	ALT > 50	0.001
	AST > 50	0.001
Severe APRI	Steatosis	0.031
AST/ALT	Steatosis	0.029

Abbreviations: ALT—alanine aminotransferase; AST—aspartate aminotransferase.

**Table 5:** Multivariate analysis of fibrosis scores for survival prediction.

Fibrosis Scores	OR	Std Error	*p*
FIB-4	1.353	0.151	0.046
AST > 50	1.02	0.009	0.037
AST/ALT	1.46	0.262	0.144
APRI	0.21	0.985	0.119
Forns	1.131	0.144	0.391

Abbreviations: ALT—alanine aminotransferase; AST- aspartate aminotransferase; OR—odds ratio; Std error—standard error.
